# Evolution in action: dissemination of *tet*(X) into pathogenic microbiota

**DOI:** 10.3389/fmicb.2013.00192

**Published:** 2013-07-10

**Authors:** Rustam I. Aminov

**Affiliations:** Faculty of Medical Sciences, University of the West IndiesKingston, Jamaica

In a recent publication by Leski et al. (2013), the authors reported the occurrence of multidrug-resistant *tet*(X)-containing bacterial strains in a hospital in Sierra Leone. Among 52 clinical isolates, 11 (21%) have been confirmed *tet*(X)-positive. All the positive strains have been isolated from urinary tract infections and identified as *Enterobacter cloacae, Comamonas testosteroni, Escherichia coli, Klebsiella pneumoniae, Delftia acidovorans, Enterobacter* sp., and other members of Enterobacteriaceae and Pseudomonadaceae (Leski et al., 2013).

The need for careful monitoring of *tet*(X) dissemination is dictated by the fact that the enzyme encoded by the gene, a flavin-dependent monooxygenase, is capable of degrading almost all tetracyclines, including the third-generation tetracycline, tigecycline (the minocycline derivative 9-tert-butyl-glycylamido-minocycline) (Yang et al., 2004; Moore et al., 2005). The US FDA approved tigecycline in 2005, and its use in the EU was authorized in 2006. Its use is approved for complicated skin and intra-abdominal infections as well as community-acquired pneumonia (http://www.accessdata.fda.gov/drugsatfda_docs/label/2010/021821s021lbl.pdf). The antibiotic is very efficient in treatment of a number of infections, including those resistant to the first- and second-generation tetracyclines (Bertrand and Dowzicky, 2012). Despite being considered as a drug of last resort, its use is steadily increasing, at least in the US (Huttner et al., 2012).

**Figure 1:**
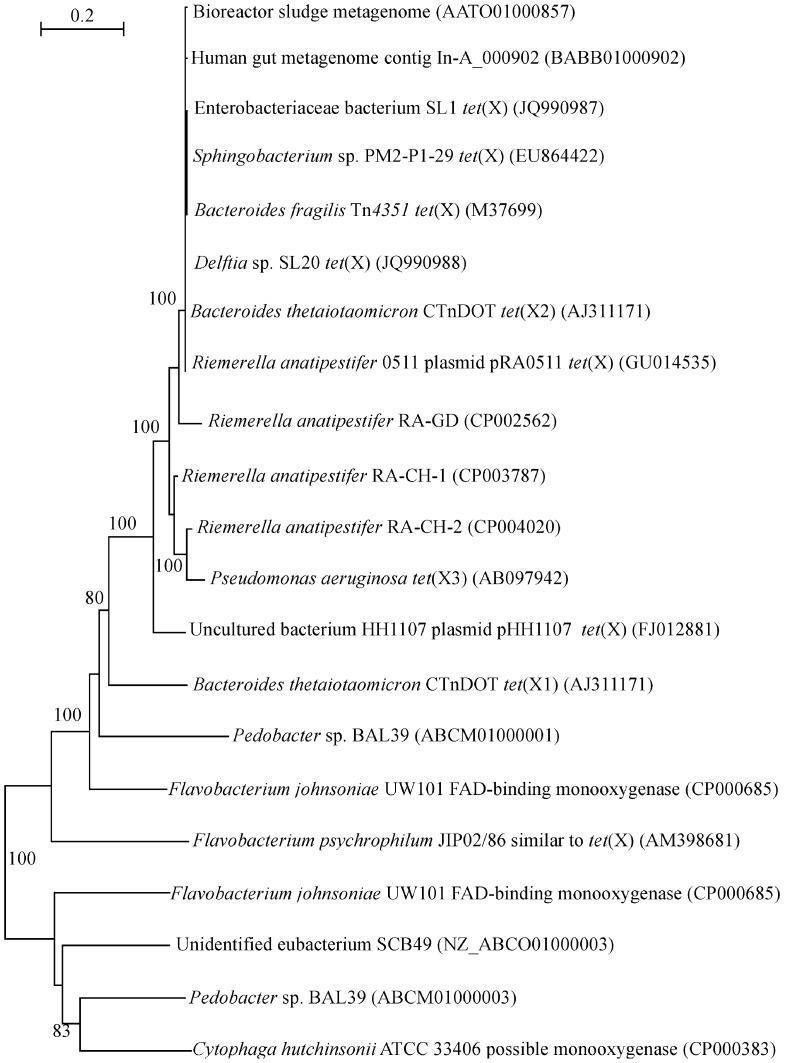
**Neighbor-joining tree of the *tet*(X) and flavin monooxygenase-encoding genes**. Numbers above each node show the percentage of tree configurations that occurred during 1000 bootstrap trials. The scale bar is in fixed nucleotide substitutions per sequence position. GenBank accession numbers of nucleotide sequences used in this analysis are given in parenthesis.

Although tigecycline resistance has not been tested at the time of isolation (Leski et al., 2013), the high frequency of *tet*(X) encountered in clinical samples signifies a worrying trend. In the previous analysis of the occurrence and phylogeny of the *tet*(X) genes it has been established that these genes can be detected in environmental DNAs and isolates as well as commensal bacteria (Aminov, 2009). Further studies have not spotted any expansion beyond these ecological niches. The presence of *tet*(X) has been detected in the human gut bacteria (de Vries et al., 2011), intestinal *Bacteroides* strains (Bartha et al., 2011), sewage treatment plants (Zhang and Zhang, 2011), and an oxytetracycline production wastewater treatment system (Liu et al., 2012). But now *tet*(X) is detected in a variety of clinical isolates and accepted human pathogens (Leski et al., 2013). The *tet*(X) sequences from this study have been added to the previous dataset (Aminov, 2009), and the phylogenetic tree has been recomputed (Figure 1). It is not surprising to see a tight clustering, with a 100% bootstrap support, of the *tet*(X) sequences from Enterobacteriaceae bacterium SL1 and *Delftia* sp. SL20 with the known *tet*(X) genes, given the high similarity of sequences within the cluster that exceeds 99%.

It is important to note here that there is no access to tigecycline (Tygacil®, Pfizer Inc.) in the hospital where *tet*(X)-positive samples were collected nor it is available through the independent pharmacies and hospital dispensaries operating in the area (Leski et al., 2013). Still, 87% of pharmacies dispense the “older” tetracyclines without prescription. As the authors suggest, this selective pressure of continuous application of tetracyclines may serve to maintain and spread *tet*(X) and other tetracycline resistance genes into pathogenic microbiota. Also, the probability of co-selection cannot be ruled out. The authors indicated the presence of mobile genetic elements in some isolates, and 10 out of 11 isolates appeared to be harboring multidrug resistance determinants.

In animal production systems, the penetration of *tet*(X) into the pathogens happened earlier. This can be demonstrated with the example of *Riemerella anatipestifer*, a causative agent of septicaemia anserum exsudativa (Segers et al., 1993). Septicaemia leads to major economic losses in duck production (Ryll et al., 2001; Sarver et al., 2005) but it also affects other bird species (Sandhu and Rimler, 1997; Hess et al., 2013). The *R. anatipestifer* strain, resistant to ampicillin, chloramphenicol, gentamicin, amikacin, tetracycline, nalidixic acid, and trimethoprim/sulfamethoxazole, was isolated in 2005 from waterfowl in Taiwan (Chen et al., 2010). It carries pRA0511 plasmid, which, in addition to two chloramphenicol acetyltransferases and a multi-drug ABC transporter permease/ATPase, also encodes TetX. The gene sequence has been incorporated into the existing dataset (Aminov, 2009) and recomputed (Figure 1). Similar to the genes from human pathogens, the gene from the poultry pathogen is confidently grouped into the *tet*(X) cluster. Three genomic sequences of *R. anatipestifer*, published (Yuan et al., 2011) or available as database entries (GenBank accession numbers CP003787 and CP004020), also carry chromosomally encoded genes similar to *tet*(X) (Figure 1). Interestingly, four other strains of *R. anatipestifer*, for which genome sequences are available (Mavromatis et al., 2011; Zhou et al., 2011; Wang et al., 2012; Yuan et al., 2013), have not yet acquired *tet*(X). No information regarding antibiotic use practices at sampling sites where *R. anatipestifer* strains have been isolated is available in the cited publications.

It seems that the use of even ‘older’ antibiotics may contribute to the resistance to newer antibiotics. There is no access to the third-generation tetracycline, tigecycline (Tygacil®, Pfizer Inc.), in the areas sampled in Sierra Leone (Leski et al., 2013). It is also highly unlikely that this expensive new antibiotic is used in duck production, most likely these are the first-generation tetracyclines. Thus the conclusion is that the selective pressure by older antibiotics drives the resistance to a newer antibiotic and contributes to the dissemination of this resistance to pathogens.

The flavoprotein monooxygenase group of enzymes is found in many metabolic pathways involved in the region-specific hydroxylation of organic substrates in all three domains of life (Harayama et al., 1992). Based on sequence similarity and 3D structural data, the enzymes are divided into six classes (van Berkel et al., 2006). Class A enzymes, to which TetX belongs, are generally involved in the degradation of phenolic compounds by *ortho*- or *para*-hydroxylation of the aromatic ring (Moonen et al., 2002).

Bacteria that carry these genes are omnipresent and can be encountered in a variety of ecosystems, including soil, aquatic ecosystems, and intestinal tract; some are opportunistic pathogens. Accordingly, the range of biochemical reactions performed by this class of enzymes is quite broad, and they may play an important role in the global carbon and nitrogen cycles (Chen et al., 2011; Wang and Shao, 2012). Interestingly, the range of metabolic activities expressed by these enzymes also includes the modification of many antibiotics. Besides the tetracylines discussed here, this range is extended to such structurally different antibiotics as rifampin (Andersen et al., 1997), mithramycin (Prado et al., 1999), griseorhodin (Li and Piel, 2002), chromomycin (Menendez et al., 2004), and auricin (Novakova et al., 2005).

The genetic context of flavin monooxygenase genes has been discussed earlier (Aminov, 2009). In brief, the majority of the genes analysed is almost uniformly associated with mobile genetic elements, including the plasmid-encoded *tet*(X) discussed here (Chen et al., 2010). The genes in this class are also highly incongruent with taxonomic positioning suggesting horizontal gene transfer events. They are also subject to frequent duplication events, which are partially illustrated here with the paralogous genes from *Flavobacterium johnsoniae* UW101 and *Pedobacter* sp. BAL39 (Figure 1).

The case of flavin monooxygenases is a vivid example demonstrating enormous adaptability of bacteria: they can freely move their protective armours amongst a variety of ecological compartments in response to yet another challenge, this time inflicted by humans in the form of antibiotic selective pressure. The global microbiota has been dealing with environmental challenges for billions of years to become sophisticated genetic engineers moving genes around with ease (Aminov, 2011). Combined with the readily available massive metabolic resources of the environmental metagenome, the microbiota seem capable of countering any kind of environmental or anthropogenic assault.

We are living in a fascinating era with technological advancements that allow us to see almost instantaneously the evolutionary events leading to the emergence of novel pathogens armed with resistance mechanisms against the most advanced antibiotics that we have been able to design. We should not underestimate the enormous genetic flexibility and the vast metabolic capabilities of the environmental microbiota. Based on our technical capabilities and knowledge acquired during the antibiotic era (Aminov, 2010), we have to make every effort, at every level possible, to preserve the power of antibiotics. Taking a bystander position in this situation is not acceptable.
